# Protocol: Comparative efficacy of 2 mL vs. 5 mL 0.375% ropivacaine in subparaneural upper trunk brachial plexus block for postoperative analgesia after arthroscopic shoulder surgery—a randomized noninferiority trial

**DOI:** 10.3389/fmed.2026.1865264

**Published:** 2026-05-28

**Authors:** Jiping Lou, Yu Zhang, Jie Chen, Shunli Diao, Chaoxu Sheng, Xiaolu Huang, Liyong Yuan, Miao Zhu

**Affiliations:** 1 Department of Anesthesiology, Ningbo Yinzhou No.3 Hospital, Ningbo, Zhejiang, China; 2 Department of Anesthesiology, Ningbo No.6 Hospital, Ningbo, Zhejiang, China; 3 Ningbo Clinical Research Center for Orthopedics, Sports Medicine & Rehabilitation, Ningbo, Zhejiang, China; 4 Department of Operating Room, Ningbo Hospital of Integrated Traditional Chinese and Western Medicine, Ningbo, Zhejiang, China

**Keywords:** brachial plexus block, hemidiaphragmatic paresis, non-inferiority, protocol, upper trunk

## Abstract

**Introduction:**

Interscalene brachial plexus block (ISB) is the first-line analgesic technique for arthroscopic shoulder surgery, but it is associated with a > 90% incidence of ipsilateral hemidiaphragmatic paresis (HDP), which severely limits its application in patients with preexisting pulmonary dysfunction. All currently available modified nerve block techniques have failed to achieve a 0% incidence of HDP while maintaining satisfactory analgesic efficacy. Our preliminary study demonstrated that subparaneural injection of 2 mL 0.375% ropivacaine at the bifurcation of the brachial plexus upper trunk provided excellent postoperative analgesia with no observed cases of HDP.

**Methods and analysis:**

This is a prospective, single-center, randomized, non-inferiority trial conducted at Ningbo No.6 Hospital, China. A total of 100 patients undergoing elective arthroscopic shoulder surgery will be randomized in a 1:1 ratio to receive either 2 mL or 5 mL of 0.375% ropivacaine via ultrasound-guided subparaneural injection at the upper trunk bifurcation. The dual co-primary endpoints are the pre-rescue Numerical Rating Scale (NRS) pain score immediately after full consciousness in the post-anesthesia care unit (PACU) (non-inferiority margin = 1 point) and the incidence of ipsilateral HDP assessed before PACU discharge. Secondary outcomes include diaphragmatic function parameters, pulmonary function, perioperative analgesic consumption, recovery indicators, and adverse events. All statistical analyses will be performed according to the pre-specified statistical analysis plan, with the intention-to-treat population as the primary analysis set.

**Clinical trial registration:**

http://www.chictr.org.cn, ChiCTR2500114139.

## Introduction

Shoulder pain is one of the most prevalent musculoskeletal disorders worldwide, affecting 7 to 34% of adults at any given time, with higher morbidity in women, elderly individuals, and manual workers (1, 2). In China, the annual prevalence of shoulder pain reaches 30 to 35% in the general adult population, and exceeds 50% in high-risk occupational groups, resulting in substantial productivity loss and medical resource consumption globally (3–6). For patients with rotator cuff injury and subacromial impingement syndrome—the leading causes of chronic shoulder pain, accounting for 44 to 78% of musculoskeletal shoulder pain cases—arthroscopic rotator cuff repair and subacromial decompression have become the mainstream surgical treatments worldwide, with over 370,000 procedures performed annually in the United States alone (7–10).

Despite the minimally invasive nature of arthroscopic shoulder surgery, patients often experience moderate to severe pain within the first 48 h postoperatively, with pain intensity comparable to that of open surgery (11). Traditionally, this postoperative pain is managed with opioid analgesics, the required dosage of which is similar to that for gastrectomy or thoracotomy (12, 13). However, opioid-based analgesia is associated with a series of adverse events including nausea, vomiting, pruritus and constipation, and even multimodal analgesia regimens fail to reduce opioid consumption significantly in patients undergoing rotator cuff surgery, driving the development of opioid-sparing regional anesthesia techniques (14–16).

Interscalene brachial plexus block (ISB) is currently the first-line opioid-sparing analgesic regimen for shoulder surgery, as it significantly reduces postoperative pain scores, improves patient satisfaction, and shortens hospital stay (17). Nevertheless, ISB has a critical and potentially life-threatening limitation: it is associated with a more than 90% incidence of ipsilateral hemidiaphragmatic paresis (HDP). [18–20]HDP leads to a 27% reduction in forced vital capacity (FVC) and forced expiratory volume in 1 s (FEV1), which is intolerable for patients with preexisting pulmonary dysfunction, who may develop life-threatening hypoxemia and respiratory distress after HDP occurs (18, 19). Paradoxically, these high-risk patients are also not candidates for general anesthesia alone, as anesthetics, analgesics, and muscle relaxants can further impair their respiratory function and worsen clinical outcomes (20, 21).

To reduce the risk of HDP, various modified nerve block techniques have been explored, including low-volume ISB, costoclavicular block, combined axillary and suprascapular nerve block and anterior shoulder capsule block. However, even low-dose ISB still has an HDP incidence of over 20%, and costoclavicular block, initially reported to have zero HDP risk, has been verified to have an HDP rate ranging from 7.5 to 50% in subsequent studies including our team’s previous work (22–24). Other aforementioned modified techniques remain clinically controversial, with multiple studies reporting insufficient pain control for arthroscopic shoulder surgery and high rates of rescue analgesia (25, 26). Recently, Zhang et al. reported that subparaneural injection of 5 mL ropivacaine at the bifurcation of the brachial plexus upper trunk achieved non-inferior analgesia compared with ISB, while reducing the HDP incidence to 16.7% (27). While this finding is promising, the 16.7% HDP risk is still unacceptable for patients with severe pulmonary impairment, as even minimal risk of HDP can lead to catastrophic clinical outcomes, following the “all-or-none” principle of HDP prevention (21). The minimal effective volume of local anesthetic for this technique, which could eliminate HDP risk while maintaining satisfactory analgesia, remains undefined.

Our team conducted a preliminary study enrolling 11 patients undergoing elective arthroscopic shoulder surgery, and found that subparaneural injection of 2 mL 0.375% ropivacaine at the upper trunk bifurcation provided excellent postoperative analgesia, with no cases of HDP observed in this small cohort. Based on these promising preliminary findings, we designed this trial to assess whether 2 mL of 0.375% ropivacaine provides non-inferior analgesic efficacy compared with 5 mL, while significantly reducing the incidence of HDP. If confirmed, the results of this trial may help define a potential ‘zero-risk dose threshold’ for this technique, and could provide a safer analgesic option for patients undergoing shoulder surgery, especially those with compromised pulmonary function.

## Methods

### Ethics and dissemination

The research protocol, informed consent form and all study-related materials of this trial have been reviewed and approved by the Ethics Committee of Ningbo No.6 Hospital (Ethics Approval No.X2025-14(K)-1). This prospective, single-center, randomized, non-inferiority trial has been prospectively registered at the Chinese Clinical Trial Registry (http://www.chictr.org.cn), with the trial registration number ChiCTR[2500114139] and registration date [December 08, 2025]. This study is conducted in strict accordance with the ethical principles of the Declaration of Helsinki, and follows the Consolidated Standards of Reporting Trials (CONSORT) guidelines for randomized controlled non-inferiority trials.

All participants will provide written informed consent prior to any study-related procedures during the preoperative anesthesia visit. All subjects reserve the right to withdraw from the study at any time without any penalty or negative impact on their routine perioperative clinical care and treatment. The personal identifiable information of all participants will be strictly encrypted and kept confidential, with access limited only to authorized research team members. All original study data, including signed informed consent forms, case report forms, ultrasound measurement records, and electronic medical records, will be securely archived and retained for at least 5 years after the completion of the study, in compliance with Good Clinical Practice (GCP) regulations and the requirements of the institutional ethics committee.

**Figure 1:**
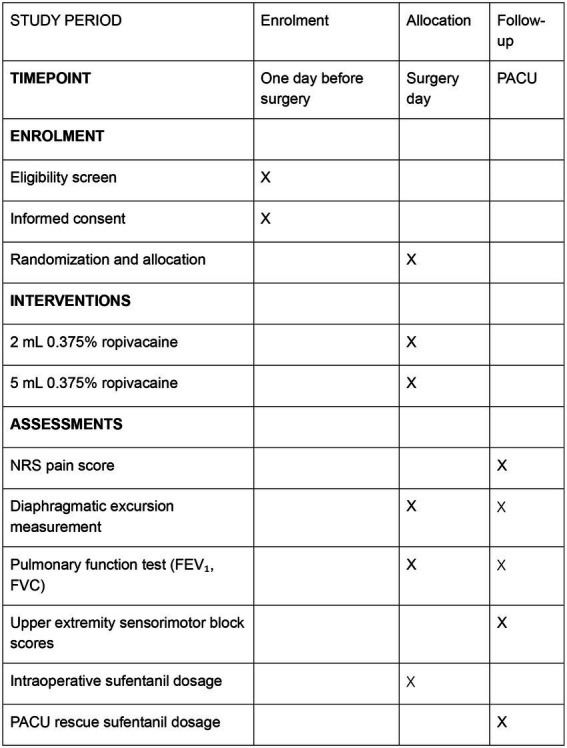
Schedule of enrolment, interventions, and assessments for the randomized controlled non-inferiority trial.

**Figure 2:**
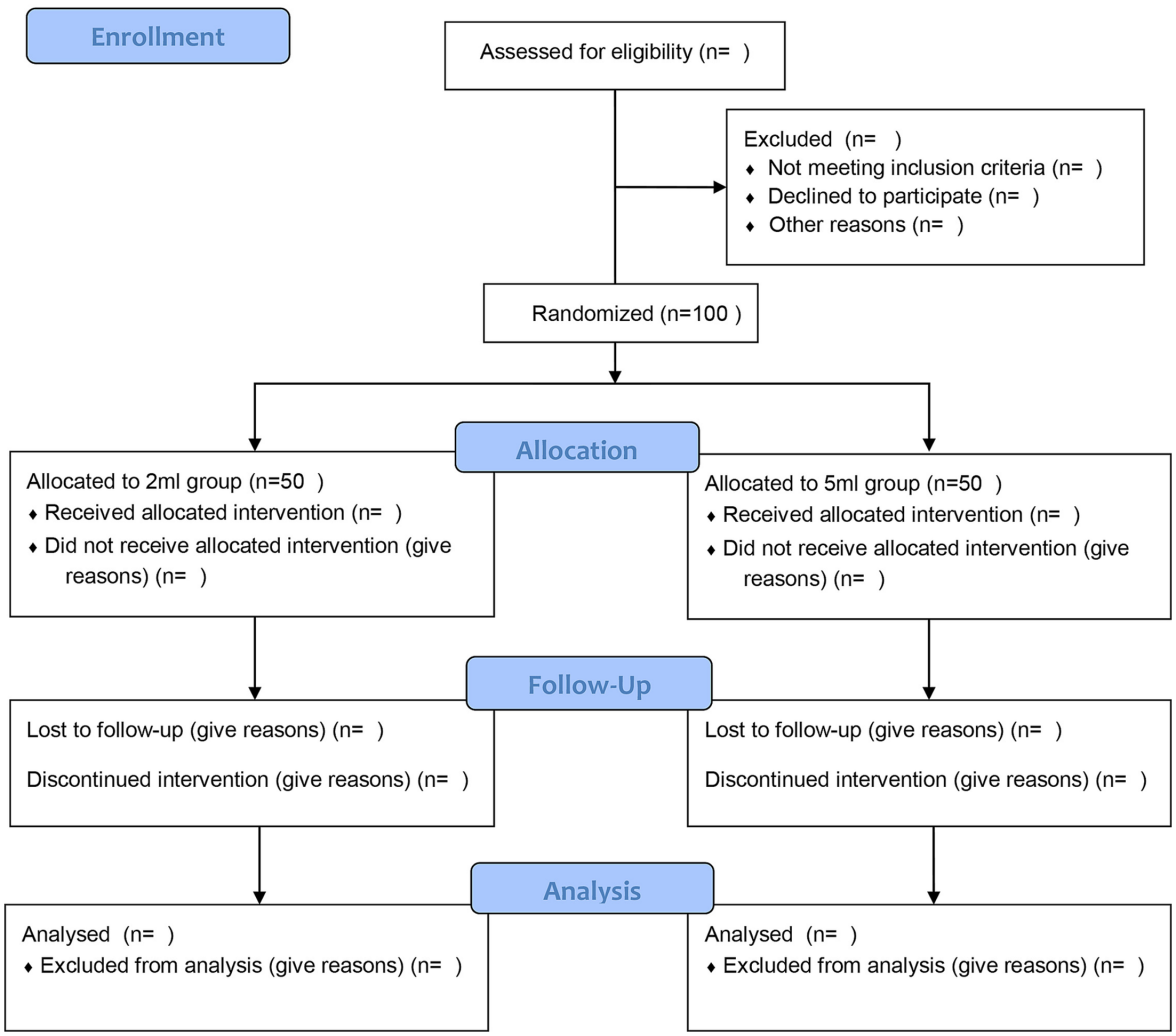
Flow chart of patient screening, eligibility assessment, randomization, and follow-up.

This protocol is written and reported in full adherence to the Standard Protocol Items: Recommendations for Interventional Trials (SPIRIT) guidelines. The detailed schedule of participant enrollment, interventions, and outcome assessments is presented in Figure 1, and the flow chart of patient screening, eligibility assessment, randomization, and follow-up is illustrated in Figure 2.

### Status and timeline

*Participant recruitment*: Recruitment of eligible participants is scheduled to initiate on January 1, 2026, and is anticipated to be completed by December 31, 2026, at the Department of Anesthesiology, Ningbo No.6 Hospital.

*Data collection*: Perioperative baseline data, intervention-related data, and primary and secondary outcome data collection will be conducted concurrently with participant enrollment, and is expected to be fully completed by December 31, 2026. Data quality verification and cross-check will be performed synchronously during the collection period to ensure the integrity and accuracy of the dataset.

*Data management and statistical analysis*: Data cleaning, database locking, and final quality audit will be completed from January 2027 to February 2027. Formal statistical analysis for the primary non-inferiority test and secondary outcome analysis will be conducted from February 2027 to June 2027, in strict accordance with the predefined statistical analysis plan.

*Results availability*: Preliminary analysis results will be available by June 2027. The final study results are expected to be fully reported and submitted for academic publication by December 2027, with the development and promotion of the standardized operation procedure initiated thereafter.

### Subjects and setting

We plan to enroll 100 patients scheduled for elective arthroscopic shoulder surgery at the Department of Anesthesiology, Ningbo No.6 Hospital, Ningbo, Zhejiang, China. Eligible participants will be randomized in a 1:1 ratio to the 2 mL group or 5 mL group, receiving 2 mL or 5 mL of 0.375% ropivacaine via ultrasound-guided subparaneural injection at the bifurcation of the brachial plexus upper trunk, respectively.

Inclusion criteria:

1. Aged 18 to 75 years
2. Scheduled for elective arthroscopic shoulder surgery under general anesthesia combined with brachial plexus block
3. American Society of Anesthesiologists (ASA) physical status I–III
4. Body mass index (BMI) ranging from 18.5 to 30 kg/m^2^
5. Fully understand the study protocol and voluntarily sign the written informed consent form before any study-related procedures

The exclusion criteria will be as follows:

1. Refusal to participate in the study or inability to provide informed consent
2. Known hypersensitivity to amide local anesthetics
3. Preexisting nerve injury or sensory abnormality in the operative upper limb
4. History of surgery in the ipsilateral shoulder or cervical region
5. Suspected infection at the puncture site of the ipsilateral shoulder or neck
6. Preexisting pulmonary disease confirmed by preoperative pulmonary function test (defined as FEV1/FVC < 70%)
7. Coagulopathy (international normalized ratio [INR] > 1.5 or platelet count < 80 × 10^9^/L)
8. Chronic pain condition requiring regular opioid medication for more than 3 months preoperatively
9. Concomitant mental, speech, or hearing impairment that hinders the completion of pain score assessment, diaphragmatic function and pulmonary function tests.

### Participants’ consent

One day prior to the surgical procedure, investigators Chaoxu Sheng will perform eligibility screening on patients slated for elective arthroscopic shoulder surgery the subsequent day. For those patients who satisfy all the inclusion criteria and none of the exclusion criteria, the investigators will disclose the study-related details to the patients and their family members or legally authorized representatives in an easy-to-understand manner, with sufficient time reserved for the patients to ask questions and confirm details.

The disclosed information encompasses the study’s primary and secondary objectives, detailed procedural steps (including preoperative baseline measurement of diaphragmatic function and pulmonary function, ultrasound-guided subparaneural brachial plexus block at the upper trunk bifurcation, standardized general anesthesia management, and postoperative outcome assessment in the post-anesthesia care unit [PACU]), potential therapeutic benefits (effective postoperative pain control with reduced risk of hemidiaphragmatic paresis and respiratory-related complications), possible adverse events (including local anesthetic allergy, nerve injury, local anesthetic toxicity, intraoperative hypotension, postoperative nausea and vomiting, and other anesthesia-related complications), and corresponding risk-mitigation strategies. The investigators will also explicitly inform the patients that the study is for scientific research purposes only, and their participation will not incur any additional medical expenses beyond routine clinical treatment.

In the event that patients express their willingness to partake in the study after fully understanding all the above information, either they or their legally authorized representatives are required to sign the written informed consent form in triplicate. One copy of the signed form will be provided to the participant or their legal representative, one will be filed in the participant’s electronic medical record, and the third will be securely archived by the research team in the study-specific locked filing cabinet, in compliance with the requirements of the institutional ethics committee.

All participants reserve the unconditional right to withdraw from the study at any time, for any reason, without any penalty, restriction, or negative impact on their routine perioperative clinical care and treatment. For participants who withdraw from the study, the research team will continue to provide standardized clinical treatment and care in accordance with clinical guidelines, and will not discriminate against or retaliate against the participant in any form. For participants who withdraw before the completion of all study assessments, the research team will not collect additional follow-up data without the participant’s explicit consent; de-identified data collected before withdrawal will be retained for statistical analysis only after obtaining ethical approval. No individual identifiable information of the participants will be used in any research report or academic publication.

### Randomization and blindness

#### Randomization procedure and allocation concealment

This study uses a blocked randomization design with a fixed block size of 4 to ensure a strict 1:1 allocation ratio throughout the entire recruitment process. This design guarantees that after every 4 subjects are enrolled, there will be exactly 2 subjects in each group, eliminating any risk of significant group imbalance even in the presence of dropouts. The randomization sequence was generated by Yu Zhang using R software (version 4.4.3) with a fixed random seed of 13,579 to guarantee full reproducibility of the allocation sequence. A total of 100 eligible patients will be randomly assigned to either the 2 mL 0.375% ropivacaine group or the 5 mL 0.375% ropivacaine group.

Allocation concealment was achieved using the sequentially numbered, opaque, sealed envelopes (SNOSE) method, which is the gold standard for allocation concealment in single-center clinical trials. Each random allocation result was recorded on a pre-printed card, which was then placed into an individual opaque, non-resealable envelope. The envelopes were numbered sequentially from 1 to 100 and sealed to prevent any prior tampering or detection of the allocation result.

Yu Zhang, who generated the randomization sequence, has no involvement in patient recruitment, intervention implementation, outcome assessment, or data analysis. The sealed envelopes are stored in a locked filing cabinet with restricted access, and only Jie Chen, the dedicated anesthesiology nurse responsible for study drug preparation, has access to the envelopes. When a patient meets all inclusion criteria and provides written informed consent, Jie Chen will open the next sequentially numbered envelope immediately before the nerve block procedure, prepare the study drug according to the allocation result, and record the envelope opening time and group assignment in a dedicated study log.

#### Unblinding procedures

Formal final unblinding of the entire study will only be performed after complete database locking (all outcome data have been collected, verified, and frozen with no further modifications permitted) and finalization of the pre-specified statistical analysis plan. The unblinding ceremony will be conducted in the presence of the independent Data Monitoring Committee (DMC), principal investigator Jiping Lou, independent statistician, Yu Zhang, and a representative of the institutional Ethics Committee.

Unblinding is permitted only when it is absolutely essential to know the group allocation for the emergency treatment of the subject, such as the occurrence of life-threatening adverse events judged to be related to the study drug. The unblinding process is to be carried out exclusively by the principal investigator Jiping Lou, with Jie Chen required to provide the original envelope allocation record for verification. A detailed written record of the reason for unblinding, time, subject information, and group allocation must be made immediately by the principal investigator and filed with the nurse’s original records. The event and unblinding details must be reported to the institutional Ethics Committee within 24 h. All other investigators and study personnel are expected to maintain the blinded state to the greatest extent possible, and the unblinded subject will be excluded from the per-protocol (PP) analysis set, while retained in the intention-to-treat (ITT) analysis set as the primary analysis population of the trial.

#### Handling of study withdrawals

No subjects who withdraw from the study for any reason will be replaced, which is fully consistent with the ITT principle. All randomized subjects, regardless of whether they complete the study or not, will be included in the primary ITT analysis. The sample size calculation for this trial has already accounted for a conservative 20% dropout rate, so the statistical power of the study will be maintained even with the expected number of withdrawals.

For subjects who withdraw before completing all primary outcome assessments, de-identified data collected before withdrawal will be retained for statistical analysis. No additional follow-up data will be collected without the subject’s explicit written consent.

#### Blinding procedures

This is a patient- and outcome assessor-blinded randomized controlled trial. The following study personnel remain fully blinded to the group allocation throughout the entire study period:

- All study participants
- The independent outcome assessor (Miao Zhu, responsible for pain score assessment, diaphragmatic function measurement, and pulmonary function testing)
- The intraoperative general anesthesia management team
- All post-anesthesia care unit (PACU) and ward nursing staff
- The data management team
- The independent statistician

Only two study personnel are aware of the group allocation: Jie Chen, the dedicated anesthesiology nurse responsible for study drug preparation and envelope management, and Shunli Diao, the anesthesiologist exclusively performing the ultrasound-guided nerve block procedure. Strict measures have been implemented to minimize potential bias introduced by these unblinded personnel:

- Complete separation of roles: Shunli Diao does not participate in any baseline or postoperative outcome assessments, patient recruitment, or data analysis
- Prohibition of any communication regarding group allocation between unblinded personnel and all blinded study staff
- Standardized preparation of study drugs in identical 5 mL syringes with no distinguishing marks
- Application of an opaque sterile dressing to the puncture site after the block procedure to maintain patient blinding

### Baseline pulmonary function test and diaphragmatic excursion measurement

Subjects sign the written informed consent form one day before surgery, and remain fully blinded to their group allocation throughout the perioperative period. No premedication is administered to any subject in the ward before surgery.

On the day of elective surgery, all subjects follow the standard preoperative fasting protocol, with 8 h of fasting for solid food and 2 h of fasting for clear liquids. After entering the operating room, a peripheral venous access is established on the non-operative upper limb of each subject, followed by a rapid infusion of 8 mL/kg lactated Ringer’s solution over 15–20 min for initial volume expansion. For patients with a history of cardiovascular disease (including heart failure, coronary artery disease, or hypertension), the infusion rate and total volume will be individually adjusted based on clinical assessment to avoid volume overload. Standard non-invasive monitoring is initiated immediately, including continuous electrocardiogram (ECG), non-invasive blood pressure (NIBP), and pulse oxygen saturation (SpO₂). Baseline vital signs (heart rate, systolic blood pressure, diastolic blood pressure, mean arterial pressure, and SpO₂) are recorded before any anesthetic or study intervention, and all intraoperative vital signs are continuously documented in the electronic anesthesia record system throughout the procedure.

Immediately after the above preoperative preparation is completed and strictly before the implementation of the study intervention (ultrasound-guided subparaneural block at the brachial plexus upper trunk bifurcation), all baseline diaphragmatic excursion and pulmonary function measurements are performed. The core purpose of these measurements is to obtain paired baseline data for the primary and secondary endpoints of this patient- and outcome assessor-blinded randomized controlled trial, so as to evaluate the differences in diaphragmatic function and pulmonary function between the 2 mL and 5 mL 0.375% ropivacaine groups after the study intervention.

All baseline and postoperative pulmonary function testing and diaphragmatic excursion measurements are independently completed in full by Miao Zhu. Prior to trial initiation, Miao Zhu has completed standardized, centralized training for ultrasound diaphragmatic function assessment and pulmonary function testing, with a verified track record of more than 200 standardized, qualified procedures, to ensure the standardization of operational processes, the repeatability of measurements, and the reliability of the collected data, and to minimize inter-observer and intra-observer variability. To strictly maintain the integrity of the patient- and outcome assessor-blinded design, Miao Zhu remains fully blinded to the group allocation of all subjects throughout the entire study period.

The entire study intervention procedure is independently completed in full by Shunli Diao, a senior anesthesiologist with extensive experience in ultrasound-guided brachial plexus block. To strictly enforce the separation of roles and avoid unblinding, Shunli Diao is prohibited from participating in any baseline measurement procedures, has no access to the baseline measurement data collected by Miao Zhu, and does not participate in postoperative outcome assessment or data statistical analysis. Shunli Diao only obtains the pre-prepared study drug corresponding to the subject’s enrollment number from Jie Chen, the dedicated anesthesiology nurse responsible for randomization management and study drug preparation, only after Miao Zhu has fully completed all baseline measurements, archived the original data, and handed over the subject to the surgical team. Shunli Diao is only aware of the injection volume of the study drug for the individual enrolled subject, and is strictly prohibited from disclosing any allocation-related information to Miao Zhu, the subject, the anesthesia management team, or the surgical team.

All measurements are performed with the subject placed in a unified, standardized semi-sitting position, with the head of the bed elevated at 30 degrees, the head maintained in a neutral position, and both upper limbs placed naturally on both sides of the body. This body position is strictly consistent between the baseline measurement and the postoperative follow-up assessment, to avoid deviations in diaphragmatic excursion and pulmonary function test results caused by postural differences, and ensure the comparability of paired pre- and post-intervention data.

For diaphragmatic excursion measurement, the anterior subcostal approach is adopted by Miao Zhu, with the liver (for subjects undergoing right-sided shoulder surgery) or the spleen (for subjects undergoing left-sided shoulder surgery) used as the acoustic window. The measurement is performed using the SONOSITE SII® ultrasound system (FUJIFILM SonoSite, Inc., Bothell, USA) equipped with a low-frequency (1 to 5 MHz) convex array probe. After clearly visualizing the continuous diaphragmatic pleural line, the probe position and angle are fixed, and the system is switched to M-mode to record the waveform of diaphragmatic movement during deep breathing. Three stable and reproducible diaphragmatic movement waveforms during deep breathing are continuously recorded for each subject, the excursion amplitude (cm) of the ipsilateral hemidiaphragm is measured for each valid waveform, and the average value of the three measurements is calculated and documented as the baseline diaphragmatic excursion value of the subject. All original ultrasound images of the measurements are archived in real time for subsequent review and data verification.

For pulmonary function testing, Miao Zhu uses a CONTEC SP80B handheld spirometer (CONTEC Medical Systems Co., Ltd., Hebei, China) with the subject remaining in the same standardized semi-sitting position. The subject wears a nose clip and holds a disposable mouthpiece, and completes forced expiration maneuvers following standardized verbal instructions, in strict accordance with international pulmonary function testing guidelines. Three valid and reproducible forced expiration measurements are completed for each subject, with invalid curves (such as those caused by insufficient expiratory time, poor initial expiratory burst, or air leakage) excluded. The forced expiratory volume in 1 s (FEV1) and forced vital capacity (FVC) of each valid measurement are recorded, and the average value of the three valid measurements is calculated and documented as the baseline FEV1 and FVC values of the subject. The baseline FEV1/FVC ratio is calculated simultaneously, and subjects with a baseline FEV1/FVC < 70% are excluded from the trial in accordance with the pre-specified exclusion criteria.

All baseline measurement data are entered into a standardized case report form (CRF) marked only with the subject’s unique study ID (no group allocation information) in real time by Miao Zhu, and then independently double-entered into the study-specific locked electronic database by two other researchers who are not involved in the measurement or intervention procedures, with cross-verification performed to ensure the integrity, accuracy and traceability of the data. Throughout the study, no communication related to the subject’s group allocation, intervention details, or baseline measurement results is allowed between Miao Zhu and Shunli Diao, to strictly maintain the blind design and eliminate any potential assessment bias.

### Ultrasound-guided subparaneural block at the brachial plexus upper trunk bifurcation

All block procedures are independently performed in full by Shunli Diao, a senior anesthesiologist with extensive experience in ultrasound-guided brachial plexus block, who has completed more than 200 standardized ultrasound-guided upper trunk block procedures prior to the trial. Shunli Diao is prohibited from participating in any baseline or postoperative outcome assessments, and only obtains the pre-prepared study drug corresponding to the subject’s enrollment number from Jie Chen, the dedicated anesthesiology nurse responsible for randomization management and study drug preparation, only after Miao Zhu has fully completed all baseline pulmonary function and diaphragmatic excursion measurements and archived the original data. Shunli Diao is only aware of the injection volume of the study drug for the individual enrolled subject, and is strictly prohibited from disclosing any allocation-related information to other study personnel, the subject, or the surgical team.

The subject is placed in a standardized supine position, with the head gently rotated to the contralateral side of the surgery, and the operative upper limb kept in a neutral position without abduction throughout the procedure, to ensure stable visualization of the brachial plexus anatomical structure. The SONOSITE SII® ultrasound system (FUJIFILM SonoSite, Inc., Bothell, USA) equipped with a high-frequency (6 to 13 MHz) linear array ultrasound probe is used for pre-scanning of the target region, following a systematic anatomical localization strategy. Scanning is commenced from the neck region to accurately locate the transverse processes of the C5 to C7 cervical vertebrae and the corresponding nerve root exit sites via cross-sectional scanning; thereafter, the probe is moved distally along the course of the brachial plexus to the supraclavicular fossa region. During the scanning process, the convergence of the C5 and C6 anterior rami to form the upper trunk of the brachial plexus is clearly visualized, followed by the trifurcation of the upper trunk into the anterior division, posterior division, and suprascapular nerve at the bifurcation point in the supraclavicular fossa. The probe is slowly tilted, slid and rotated to adjust the ultrasound beam incident angle, to obtain the optimal ultrasound view where the anterior division, posterior division, and suprascapular nerve of the upper trunk are arranged in a triangular or linear pattern within the same intact paraneural sheath.

After obtaining the optimal ultrasound view, the puncture site is disinfected and sterile drapes are applied following routine clinical sterile requirements for regional anesthesia. Local infiltration anesthesia is performed at the puncture site with 1 mL of 1% lidocaine solution. A 22 G × 50 mm nerve block needle is connected to a syringe pre-filled with the study drug (0.375% ropivacaine, 2 mL for the intervention group and 5 mL for the control group, prepared by Jie Chen in advance according to the randomization allocation). The in-plane technique is used for needle advancement, with the needle inserted from the lateral to medial direction at an angle of 30° to 45° relative to the skin (27, 28). The entire length of the needle shaft is continuously visualized under real-time ultrasound guidance throughout the advancement process.

The needle tip is advanced under continuous ultrasound monitoring to ensure accurate placement within the paraneural sheath at the bifurcation point of the upper trunk, without penetrating the nerve fascicles or violating the integrity of the paraneural sheath. After confirming the correct needle tip position, the study drug is injected into the paraneural sheath, with intermittent aspiration performed every 0.5 mL during injection to rule out intravascular or intraneural injection. The spread of the local anesthetic within the paraneural sheath is continuously monitored via real-time ultrasound throughout the injection process, to ensure circumferential spread of the drug around the three target nerve branches of the upper trunk, with no excessive diffusion toward the anterior scalene muscle or phrenic nerve region. If the subject reports paresthesia or abnormal muscle contraction during the puncture and injection process, the needle tip is immediately withdrawn, and the puncture direction is adjusted to avoid nerve injury. No additional local anesthetic is administered to any subject during the entire procedure, beyond the pre-specified volume of the study drug according to the randomization group.

Upon completion of the injection, the puncture site is covered with an opaque sterile dressing. Shunli Diao leaves the operating room immediately after the procedure, and does not participate in the subsequent general anesthesia management, intraoperative care, or postoperative outcome assessment of the subject. Any adverse events during the block procedure, including inadvertent vascular puncture, local anesthetic toxicity, persistent paresthesia, or bleeding at the puncture site, are fully documented in the subject’s case report form and electronic medical record in real time.

### Anesthesia management

#### General anesthesia induction

After the completion of the ultrasound-guided subparaneural injection at the brachial plexus upper trunk bifurcation (the study intervention). The induction regimen consists of intravenous propofol (2–3 mg/kg) and sufentanil (5 μg); the sufentanil is administered as adjuvant analgesia to complement the preoperative nerve block, ensuring adequate analgesia during induction and initial surgical stimulation.

In strict accordance with the study protocol, no muscle relaxant is administered to any subject during the entire anesthesia induction and maintenance period, to avoid any interference with the postoperative assessment of upper limb sensorimotor block and diaphragmatic motor function. No additional sedative or analgesic drugs are administered during the induction phase, except for the study intervention and the aforementioned induction agents, to eliminate confounding factors that may affect the primary and secondary outcome assessments.

After the induction of general anesthesia, a laryngeal mask airway (LMA) is inserted to manage the patient’s airway. Correct positioning of the LMA is confirmed by bilateral equal breath sounds on auscultation, stable end-tidal carbon dioxide (EtCO_2_) waveform, and no air leakage during mechanical ventilation. Mechanical ventilation is conducted in pressure-controlled mode with standardized settings: maximum airway pressure limited to 15 cm H₂O, respiratory rate set at approximately 11 breaths per minute, and inspiratory-to-expiratory ratio of 1:2. Ventilation parameters are adjusted only to maintain EtCO₂ between 35 and 45 mmHg, ensuring respiratory stability while minimizing the risk of barotrauma.

All anesthetic agents administered during the induction phase, including the specific dose, administration time, and any adverse events during induction (such as hypotension, bradycardia, allergic reaction, or airway complications), are accurately recorded in the electronic anesthesia record sheet by the attending anesthesiologist. The anesthesiologist performing the induction is strictly prohibited from inquiring about the group allocation of the subject, and all communication related to the study intervention is restricted to Shunli Diao and Jie Chen, the dedicated anesthesiology nurse responsible for study drug preparation.

#### General anesthesia maintenance

General anesthesia maintenance is performed by the same attending anesthesiologist responsible for induction, who remains fully blinded to the subject’s group allocation throughout the procedure. Consistent with the induction protocol, no neuromuscular blocking agents are administered at any point during the maintenance phase to avoid confounding interference with the core trial outcomes of postoperative diaphragmatic motor function and upper limb sensorimotor block assessments.

The primary analgesia for the procedure is provided by the preoperative study intervention (ultrasound-guided subparaneural block at the brachial plexus upper trunk bifurcation), and the following standardized stepwise protocol is strictly enforced for all intraoperative hemodynamic fluctuations related to surgical stimulation: When the subject’s intraoperative heart rate or mean arterial pressure (MAP) exceeds 20% of the preoperative baseline value, the end-tidal concentration of inhaled sevoflurane is first increased to 3%; if the heart rate and blood pressure do not return to the baseline range within 5 min, an intravenous bolus of 2.5 μg sufentanil is administered, with repeated doses of 2.5 μg sufentanil permitted if the hemodynamic response remains uncontrolled after each dose. The total dose of sufentanil administered intraoperatively is accurately recorded for each subject as a key secondary outcome of the trial.

Sedation and hypnosis are maintained with inhaled sevoflurane (end-tidal concentration 1.5–3% in 50% oxygen/air mixture). No additional intravenous sedatives are administered unless clinically required for refractory hemodynamic instability, with all such deviations fully documented as protocol violations. Mechanical ventilation is consistent with the settings described in General anesthesia induction, without modifications, and continues until the end of the surgery.

Other intraoperative hemodynamic abnormalities are managed per standardized institutional guidelines: hypotension (MAP ≥20% below preoperative baseline) is initially managed with a 250 mL lactated Ringer’s bolus, followed by 8 μg norepinephrine boluses if persistent; clinically significant bradycardia (heart rate ≤45 bpm with hemodynamic instability) is treated with intravenous atropine 0.25–0.5 mg. Fluid management follows a goal-directed protocol, with maintenance lactated Ringer’s infusion at 4–6 mL/kg/h and additional boluses titrated to intraoperative blood loss and hemodynamics. Core body temperature is maintained at 36.0–37.5 °C using a forced-air warming system throughout the procedure.

Standard continuous monitoring is maintained throughout the maintenance phase, including three-lead ECG, non-invasive blood pressure measured at 5-min intervals, continuous SpO₂, EtCO₂, end-tidal sevoflurane concentration, and nasopharyngeal temperature. All intraoperative data, including total doses of anesthetic, analgesic and vasoactive agents, anesthesia and surgery duration, fluid balance, adverse events, and protocol deviations, are fully documented in the electronic anesthesia record system, and subsequently extracted by blinded independent investigators for statistical analysis.

#### General anesthesia recovery

At the completion of the surgical procedure, the inhaled sevoflurane is discontinued immediately, and the fresh gas flow is increased to 6 L/min to accelerate the washout of the anesthetic agent from the patient’s body. Mechanical ventilation is maintained with settings consistent with those described in the general anesthesia induction section, without modifications, until spontaneous breathing is fully restored.

Extubation of the LMA is performed only when all the following extubation criteria are met: the patient regains full consciousness and can follow verbal commands; spontaneous breathing is stable, with a tidal volume ≥ 6 mL/kg predicted body weight and respiratory rate of 10–20 breaths per minute; EtCO₂ is maintained between 35 and 45 mmHg; the gag and cough reflexes are fully recovered; heart rate and mean arterial pressure remain within 20% of the preoperative baseline; and SpO₂ ≥ 95% is maintained on room air. After LMA removal, the patient is given supplemental oxygen via a face mask at 4 L/min, and transferred to the PACU accompanied by the attending anesthesiologist responsible for anesthesia management.

Upon arrival in the PACU, standard continuous monitoring is initiated immediately, including three-lead ECG, NIBP measured at 5-min intervals, continuous SpO₂, and respiratory rate monitoring. The patient’s level of consciousness, respiratory status, surgical site condition, and adverse events are assessed and documented every 15 min by PACU nursing staff who remain fully blinded to the subject’s group allocation throughout the recovery period.

Pain assessment is performed immediately after the patient regains full consciousness and can accurately report discomfort. Pain intensity is scored based on the patient’s self-reported arm or shoulder discomfort using the 11-point Numerical Rating Scale (NRS), where 0 represents no pain and 10 indicates the greatest anguish imaginable. All recorded NRS scores are the pre-rescue analgesia values. If the patient’s NRS score is greater than 3, an intravenous bolus of 5 μg sufentanil is administered, and this administration is repeated as required to achieve adequate pain control. The exact time, dose, and number of fentanyl boluses administered in the PACU are fully documented in the electronic anesthesia record system.

Discharge from the PACU is only permitted when the patient meets all the following criteria: the Aldrete recovery score is ≥ 9 points; core body temperature is maintained at 36.0–37.5 °C; hemodynamic and respiratory status are stable for at least 30 min without supplemental oxygen support; pain is well controlled with a NRS pain score ≤ 3; no severe adverse events such as persistent nausea and vomiting, dyspnea, or neurological abnormalities are observed; the required postoperative pulmonary function and diaphragmatic excursion assessments have been fully completed; and the PCA pump has been correctly initiated. All recovery-related data, including emergence time, LMA extubation time, PACU stay duration, vital signs during recovery, adverse events, and rescue analgesia administration, are fully documented in the electronic anesthesia record system and study-specific case report form (CRF) in real time.

#### Postoperative pulmonary function test and diaphragmatic excursion measurement

All postoperative pulmonary function and diaphragmatic excursion measurements are independently completed by Miao Zhu, the same investigator who performed the preoperative baseline assessments, to eliminate inter-operator variability and ensure the consistency and comparability of paired pre- and post-intervention data.

The measurements are performed when the subject is fully awake, has stable spontaneous breathing, and can accurately follow standardized verbal instructions, strictly before discharge from the PACU. The subject’s body position, equipment used, operational procedures, quality control standards, and data calculation methods for the measurements are completely consistent with those used for the preoperative baseline assessments.

Hemidiaphragmatic paralysis (HDP) is defined as a decrease in postoperative diaphragmatic excursion of ≥75% compared with the baseline value, or the presence of paradoxical diaphragmatic movement during breathing. The incidence of HDP is recorded as one of the co-primary endpoints of the trial. The absolute and percentage changes in diaphragmatic excursion, forced expiratory volume in 1 s (FEV1), and forced vital capacity (FVC) from baseline to postoperative measurement are calculated for each subject as key secondary endpoints of the trial.

All postoperative measurement data are entered into a standardized CRF marked only with the subject’s unique study ID in real time, and independently double-entered into the study-specific locked electronic database by two other researchers not involved in the measurement or intervention procedures, with cross-verification performed to ensure the integrity, accuracy and traceability of the paired data.

#### Postoperative pain control

All primary and secondary outcome data of this trial are fully collected during the PACU stay. Therefore, no standardized postoperative analgesia protocol is enforced after PACU discharge, and no additional data will be collected beyond the PACU period.

Postoperative analgesia after PACU discharge is implemented entirely according to the patient’s autonomous preference. Patients may choose either intravenous patient-controlled analgesia (PCA) or on-demand bolus analgesia administered on the ward. The specific medication for on-demand bolus analgesia is determined by the attending on-duty physician based on clinical practice guidelines and individual patient conditions.

### Data collection and management

All data collection and management processes of this trial are performed in strict accordance with the Good Clinical Practice (GCP) guidelines, the ethical principles of the Declaration of Helsinki, and the study protocol approved by the Ethics Committee of Ningbo NO.6 Hospital.

#### Data management

All study data are recorded, managed, and stored centrally via the Research Manager system (ResMan, http://www.medresman.org.cn), a web-based clinical trial management public platform certified by the Chinese Clinical Trial Registry, with complete functions for electronic case report form (eCRF) design, data entry, logical verification, audit trail, and database locking, fully complying with the regulatory requirements for clinical trial data management in China.

##### Data management governance and role allocation

An independent data management team consisting of two trained research assistants and one designated data manager is established for this trial. All members of the data management team have no involvement in the implementation of the study intervention, perioperative anesthesia management, baseline or postoperative outcome assessments, and randomization sequence generation, and remain fully blinded to the subject’s group allocation throughout the study. The specific responsibilities are clearly divided as follows:

- The designated data manager is responsible for the overall design of the eCRF in the ResMan system, database establishment, hierarchical access control setting, and supervision of the entire data management process;
- The two independent research assistants are responsible for independent dual data entry, cross-verification, and discrepancy resolution in the ResMan system, with no communication with the study intervention anesthesiologist, outcome assessor, or study drug administrator regarding any content related to group allocation;
- Jie Chen, the dedicated anesthesiology nurse responsible for randomization sequence management and study drug preparation, is the only personnel with access to the complete randomization allocation list, and is prohibited from participating in any data management, data entry, or statistical analysis procedures. The randomization allocation list is stored in a separate encrypted module in the ResMan system, which will not be accessed by any personnel until the database is formally locked.

##### Case report form (CRF) design and standardization

A standardized, study-specific eCRF is developed in the ResMan system in accordance with the study protocol, covering all data points required for the trial’s primary and secondary endpoints, with a unified coding standard and filling specification. The eCRF includes the following modules: subject screening and eligibility confirmation, written informed consent documentation, baseline demographic and clinical characteristics, baseline vital signs and pulmonary function/diaphragmatic excursion measurements, study intervention details, perioperative general anesthesia related data, intraoperative and postoperative analgesic drug consumption, postoperative primary and secondary outcome assessments, postoperative follow-up data, adverse events and serious adverse events recording, protocol deviations, and subject withdrawal or loss to follow-up documentation.

Prior to the formal initiation of the trial, the eCRF is pre-tested and validated through the pre-trial data to ensure the rationality, comprehensiveness, and operability of the form. Version control is implemented for all eCRF modifications in the ResMan system, with any changes documented in writing and approved by the principal investigator and the data management team. All eCRFs are marked only with the subject’s unique study identification number (ID), with no information that may indicate the subject’s group allocation.

##### Data entry, verification, and discrepancy resolution

A dual independent data entry system is implemented in the ResMan system for all study data. After the completion of each subject’s full follow-up, the two independent research assistants enter the data from the original paper records and archived documents into the ResMan database separately, without any communication during the entry process. After dual entry is completed, the system automatically performs cross-verification to identify inconsistent data entries and generates a discrepancy report.

For any data discrepancies, missing values, or data that do not meet the pre-specified logical rules, the data management team generates a data query form in the ResMan system. The query form is sent to the corresponding investigator for source data verification and correction, with all query processes, correction content, and supporting documents fully documented and archived in the system. Source data verification (SDV) is performed for 100% of the co-primary endpoint data (incidence of hemidiaphragmatic paralysis and PACU resting NRS pain score), and no less than 30% of secondary endpoint data, baseline data, and adverse event data, to ensure that the data in the ResMan database is completely consistent with the original medical records, anesthesia record system, ultrasound image archives, and follow-up records.

##### Database access control and lock procedure

Strict hierarchical access control is set for the ResMan database: only the designated data manager has full administrative access to the database, the two research assistants only have data entry access without modification or deletion permissions, and all other study personnel have no access to the database. The system automatically generates an immutable audit trail for all operations in the database, recording the operator, operation time, details of data entry/modification/deletion (including pre-modification and post-modification values plus reasons for changes), and permission changes, to ensure full traceability of all data operations.

After all subjects have completed the study procedures, all data entry, verification, discrepancy resolution, and SDV are fully completed, and no further data updates, corrections, or additions are expected, the database will be formally locked in a blind state in the ResMan system. The database lock procedure is jointly completed by the principal investigator, data manager, independent statistician, and ethics committee representative. A written database lock record is signed and archived by all relevant personnel. Once the database is locked, no modifications to the data are allowed, except for the correction of critical data errors identified after unblinding, which requires a written application, detailed explanation of the reasons, and approval from the institutional ethics committee, with all correction processes fully documented and traceable. Formal unblinding and statistical analysis will only be performed after the database is completely locked.

##### Data storage, confidentiality, and retention

All original study data, including signed informed consent forms, paper CRFs, original electronic anesthesia records, archived ultrasound images, pulmonary function test reports, follow-up records, and adverse event documentation, are archived in a dedicated locked file cabinet in the study center, with access restricted only to authorized study personnel. Electronic data in the ResMan system are encrypted and stored on the platform’s dedicated server, with regular automatic backups to ensure data security and prevent data loss.

All study data are de-identified, with the subject’s personal identifiable information replaced by a unique study ID, to protect the subject’s privacy in strict accordance with the Personal Information Protection Law of the People’s Republic of China and relevant international privacy regulations. All study data will be retained for at least 5 years after the completion of the study and the publication of the study results, in compliance with GCP regulations and the requirements of the institutional ethics committee.

##### Data quality assurance and monitoring

A full-process data quality control system is established throughout the study. Before the formal initiation of the trial, unified standardized training is conducted for all study personnel involved in data collection, including the outcome assessor, anesthesia management team, PACU nursing staff, and data management team, to ensure that all personnel fully understand the study protocol, eCRF filling specifications, and data collection standards in the ResMan system.

Regular on-site monitoring is performed by an independent clinical research associate (CRA) who is not involved in the study implementation, at least once a month during the subject enrollment period. The monitoring content includes the compliance of the study implementation with the protocol, the integrity and authenticity of the eCRF in the ResMan system, the consistency between the eCRF and the source data, the management of the randomization list and study drugs, and the recording and reporting of adverse events. All monitoring findings, corrective actions, and follow-up results are fully documented in the monitoring report. All protocol violations and deviations are recorded, classified, and reported to the institutional ethics committee in a timely manner, with the impact on the study results evaluated and documented.

### Data collection

All study data are collected at pre-specified time points in accordance with the study protocol, with the specific collection content as follows:

1. *Screening and baseline data*: Subject demographic information (age, gender, height, weight, BMI), ASA physical status classification, preoperative comorbidities (hypertension, diabetes, cardiovascular disease, chronic pulmonary disease, etc.), past medical history (ipsilateral shoulder or cervical surgery history, chronic pain history, allergy history), preoperative vital signs (heart rate, blood pressure, SpO₂), preoperative laboratory test results (hemoglobin, platelet count, coagulation function, liver and kidney function), and preoperative pulmonary function and diaphragmatic excursion baseline measurements (FEV1, FVC, FEV1/FVC ratio, ipsilateral diaphragmatic excursion value).
2. *Intraoperative data*: general anesthesia related data (induction and maintenance drug dosage, anesthesia duration, surgery duration, laryngeal mask airway extubation time, emergence time), intraoperative hemodynamic parameters, total dosage of intraoperative analgesic and vasoactive drugs, intraoperative fluid balance (infusion volume, blood loss, urine output), and any protocol deviations or adverse events during the operation.
3. *Post-anesthesia care unit (PACU) data*: Pre-rescue analgesia NRS pain score assessed immediately after the patient regains full consciousness from general anesthesia (before rescue analgesia in PACU), postoperative diaphragmatic excursion and pulmonary function measurements, PACU stay duration, rescue analgesia administration details, emergence and recovery related indicators, and any adverse events during the PACU stay.
4. *Safety and compliance data*: Detailed records of all perioperative adverse events and serious adverse events, including onset time, severity, causal relationship with the study intervention, treatment measures, and final outcome; detailed records of subject withdrawal, loss to follow-up, and protocol deviations, including specific reasons and corresponding handling measures.

### Outcomes

#### Primary outcomes

This trial adopts a dual co-primary endpoint design to simultaneously verify the non-inferiority of analgesic efficacy and the superiority of diaphragmatic safety of the 2 mL local anesthetic regimen:

1 Pre-rescue analgesia NRS pain score assessed immediately after the patient regains full consciousness in the PACU

- *Evaluation tool*: 11-point NRS (0 = no pain, 10 = the worst imaginable pain)
- *Evaluation time*: Immediately after full consciousness and ability to accurately report discomfort, strictly before any rescue analgesia administration
- *Study objective*: To test the non-inferiority of 2 mL 0.375% ropivacaine compared with 5 mL 0.375% ropivacaine in postoperative analgesic efficacy, with a predefined non-inferiority margin of 1 point on the NRS scale.

2 Incidence of ipsilateral hemidiaphragmatic paralysis (HDP) assessed before discharge from the PACU

- *Definition*: Complete paresis: A reduction in diaphragmatic movement of more than 75%, no movement, or paradoxical movement. Partial paresis: A reduction in diaphragmatic movement between 25 and 75%.
- *Evaluation tool*: Ultrasound measurement of diaphragmatic excursion via the anterior subcostal approach (M-mode)
- *Evaluation time*: Strictly before discharge from the PACU, performed by the same blinded investigator who conducted the baseline measurements
- *Study objective*: To test the superiority of 2 mL 0.375% ropivacaine compared with 5 mL 0.375% ropivacaine in reducing the risk of HDP.

#### Secondary outcomes

1 Diaphragmatic function parameters

- Absolute value of ipsilateral diaphragmatic excursion before discharge from the PACU
- Absolute and percentage changes in ipsilateral diaphragmatic excursion from preoperative baseline to PACU discharge

2 Pulmonary function parameters

- Absolute values of forced expiratory volume in 1 s (FEV1) and forced vital capacity (FVC) before discharge from the PACU
- Absolute and percentage changes in FEV1 and FVC from preoperative baseline to PACU discharge

3 Upper extremity sensorimotor block characteristics

- Sensory block scores of the radial, median, ulnar, musculocutaneous and axillary nerves before discharge from the PACU (0 = no block; 1 = analgesia, intact touch sensation but absent cold sensation; 2 = anesthesia, absent touch sensation)
- Motor block scores of the radial, median, ulnar, musculocutaneous and axillary nerves before discharge from the PACU (0 = normal movement; 1 = partial paralysis; 2 = complete paralysis)

4 Perioperative analgesic drug consumption

- Total intraoperative sufentanil dosage
- Total sufentanil dosage administered for rescue analgesia in the PACU

5 Recovery-related indicators

- Total time interval from completion of nerve block to PACU discharge
- Surgical duration
- PACU stay duration
- Postoperative hospital length of stay

6 Safety indicators

- Incidence of all perioperative adverse events, including nausea, vomiting, pruritus, hypotension, bradycardia, local anesthetic toxicity, nerve injury, Horner syndrome, hoarseness and dyspnea
- Incidence of serious adverse events related to the study intervention, including severe respiratory distress, anaphylactic reaction and permanent neurological deficit
- Incidence of protocol deviations and subject withdrawal/loss to follow-up, with detailed documentation of specific reasons

#### Sample size calculation

The sample size calculation is based on the dual co-primary endpoints of this trial: the non-inferiority of analgesic efficacy (PACU pre-rescue NRS pain score) and the superiority of diaphragmatic safety (incidence of hemidiaphragmatic paralysis, HDP). All calculations are performed using PASS 15.0.5 software (NCSS, LLC, Kaysville, UT, USA) with a one-sided significance level (*α*) of 0.025 and a statistical power (1-*β*) of 0.80, consistent with the design of randomized non-inferiority trials in anesthesiology.

##### Sample size calculation based on analgesic efficacy non-inferiority endpoint

The primary non-inferiority endpoint is the difference in pre-rescue NRS pain scores between the 2 mL group and the 5 mL group immediately after full consciousness in the PACU. The predefined non-inferiority margin is 1 point on the 11-point NRS scale, which is widely accepted as the minimal clinically important difference (MCID) for postoperative pain assessment in orthopedic surgery. This margin was also used in previous similar trials comparing different volumes of local anesthetic for brachial plexus block.

Based on the results of our preliminary study (*n* = 11) and previously published literature, the standard deviation (SD) of NRS pain scores in patients undergoing arthroscopic shoulder surgery with upper trunk brachial plexus block is estimated to be 1.6. Using the two-sample Z-test for non-inferiority with pooled variance, the required sample size per group is calculated as follows:

$$n=\frac{2{\left(Z_{1-\alpha }+Z_{1-\beta }\right)}^{2}\sigma^{2}}{\delta^{2}}$$

Where $Z_{1-\alpha }=1.96$ (one-sided *α* = 0.025), $Z_{1-\beta }=0.84$ (power = 0.80), σ=1.6 , and δ=1 (non-inferiority margin). Substituting the values yields a required sample size of 40 patients per group.

##### Sample size calculation based on HDP incidence superiority endpoint

The primary superiority endpoint is the difference in the incidence of ipsilateral HDP between the two groups. Based on the study by Zhang et al. (27), the incidence of HDP in patients receiving 5 mL 0.375% ropivacaine via subparaneural injection at the upper trunk bifurcation is 16.67%. Our preliminary study (n = 11) showed that no HDP occurred in patients receiving 2 mL 0.375% ropivacaine via the same technique, leading to our core hypothesis that the HDP incidence of the 2 mL regimen is infinitely close to 0.

However, the PASS 15.0.5 software does not allow setting the group proportion to 0 in the two-sample Z-test for superiority of proportions. To accurately reflect our preliminary findings and satisfy the software’s calculation requirements, we set the HDP incidence in the 2 mL group to an extremely small value of 1 × 10^−20^, which is mathematically indistinguishable from 0 for all practical clinical purposes.

Using the two-sample Z-test for superiority of proportions with pooled variance, the required sample size per group is calculated as follows:

$$n=\frac{{\left(Z_{1-\alpha }+Z_{1-\beta }\right)}^{2}(p_{1}(1-p_{1})+p_{2}(1-p_{2}))}{{\left(p_{1}-p_{2}\right)}^{2}}$$

Where $p_{1}=0.166667$ (HDP incidence in the 5 mL group), $p_{2}=1\times 10^{-20}$ (estimated HDP incidence in the 2 mL group), $Z_{1-\alpha }=1.96$ , and $Z_{1-\beta }=0.84$ . Substituting the values yields a required sample size of 40 patients per group.

##### Final sample size determination

The larger sample size requirement from the two co-primary endpoints is 40 patients per group. To account for potential subject withdrawal or loss to follow-up during the trial, we will recruit additional subjects to ensure that the final ITT analysis set includes at least 40 patients per group. Considering a conservative dropout rate of 20%, we will recruit a total of 100 patients (50 patients per group) for this trial.

Any subjects who withdraw from the study before completing all primary outcome assessments will be replaced by newly enrolled subjects in strict sequential order to maintain the integrity of the randomization sequence and ensure the required statistical power.

#### Statistical analysis

All statistical analyses will be performed in strict accordance with the pre-specified statistical analysis plan (SAP) finalized before database locking. The statistical software used will be IBM SPSS Statistics 25.0.01 (IBM Corp., Armonk, NY, USA). A two-tailed *p* value < 0.05 will be considered statistically significant, except for the one-sided tests specified for the non-inferiority and superiority primary endpoints.

##### Definition of analysis populations

Three distinct analysis populations will be defined and used for different analytical purposes:

(1) Intention-to-Treat (ITT) population (primary analysis population)

The ITT population includes all randomized subjects who have received at least one dose of the study intervention (2 mL or 5 mL 0.375% ropivacaine). This population will be used for the primary analyses of both co-primary endpoints (analgesic efficacy non-inferiority and HDP incidence superiority), as it preserves the randomization-generated group balance and minimizes selection bias, providing the most conservative and generalizable results.

(2) Per-Protocol (PP) population (sensitivity analysis population)

The PP population includes subjects who strictly adhere to the study protocol and meet all of the following criteria:

- Received the full assigned study intervention with no wrong-group medication administration
- Completed all primary outcome assessments (pre-rescue NRS pain score and diaphragmatic excursion measurement in the PACU)
- Had no major protocol violations that could impact the interpretation of primary outcomes, including:

- Preoperative use of prohibited analgesic or sedative medications
- Unplanned additional local anesthetic administration during the procedure
- Incomplete blinding due to accidental disclosure of group allocation
- Loss to follow-up before completion of primary outcome assessments

The PP population will be used for sensitivity analyses to verify the robustness of the ITT results. If the ITT and PP analyses yield consistent conclusions, the primary findings will be considered robust.

3 Safety analysis population.

The safety analysis population includes all randomized subjects who have received at least one dose of the study intervention. This population will be used for all safety-related analyses, including the incidence of adverse events and serious adverse events.

##### Handling of missing data

- For primary outcome data missing due to subject withdrawal or loss to follow-up, multiple imputation (MI) will be performed using 5 independent imputation cycles. Predictors for imputation will include baseline demographic and clinical characteristics (age, gender, BMI, ASA physical status), intraoperative factors (surgery duration, anesthesia duration, total sufentanil dosage), and available partial outcome data. A fixed random seed (12345) will be set to ensure reproducibility of the imputation results.
- For secondary outcome data with missing values, complete-case analysis will be performed, and the proportion of missing data will be reported.
- No imputation will be performed for safety data; all reported adverse events will be included in the safety analysis regardless of subject withdrawal status.

##### Analysis of primary outcomes

(1) Non-inferiority analysis of analgesic efficacy (pre-rescue NRS pain score)

The primary non-inferiority endpoint is the difference in pre-rescue NRS pain scores between the 2 mL group and the 5 mL group immediately after full consciousness in the PACU. The mean difference (MD) and its one-sided 97.5% confidence interval (CI) will be calculated using the two-sample t-test for independent samples, assuming equal variances.

*Non-inferiority judgment criterion*: The 2 mL regimen will be considered non-inferior to the 5 mL regimen in terms of analgesic efficacy if the upper bound of the one-sided 97.5% CI for the mean difference (2 mL group minus 5 mL group) is less than the predefined non-inferiority margin of 1 point.

(2) Superiority analysis of diaphragmatic safety (HDP incidence)

The primary superiority endpoint is the difference in the incidence of ipsilateral HDP between the two groups. The risk difference (RD) and its one-sided 97.5% CI will be calculated using the two-sample Z-test for proportions.

*Superiority judgment criterion*: The 2 mL regimen will be considered superior to the 5 mL regimen in terms of diaphragmatic safety if the lower bound of the one-sided 97.5% CI for the risk difference (5 mL group minus 2 mL group) is greater than 0.

##### Analysis of secondary outcomes

- *Continuous variables*: Normally distributed data (e.g., diaphragmatic excursion, FEV1, FVC, emergence time, PACU stay duration) will be presented as mean ± standard deviation (SD), and between-group differences will be analyzed using the two-sample t-test. Non-normally distributed data (e.g., analgesic drug consumption, postoperative hospital length of stay) will be presented as median (interquartile range, IQR), and between-group differences will be analyzed using the Mann–Whitney U test.
- *Categorical variables*: Data will be presented as counts (percentages). Between-group comparisons will be performed using the chi-square (χ^2^) test for large sample sizes or Fisher’s exact test for cell counts < 5.
- *Ordinal variables* (e.g., sensory and motor block scores): Between-group differences will be analyzed using the Mann–Whitney U test.

##### Multiplicity adjustment

- No multiplicity adjustment will be performed for the two co-primary endpoints, as they address distinct and independent research questions (analgesic efficacy vs. diaphragmatic safety), and both must be statistically significant to declare the overall success of the trial.
- All secondary outcomes are exploratory in nature and will not undergo multiplicity adjustment. Results will be presented with point estimates and 95% CIs, and *p* values will be interpreted descriptively rather than as definitive hypothesis tests.

##### Sensitivity analyses

To verify the robustness of the primary findings, the following sensitivity analyses will be performed:

1. Re-analysis of both co-primary endpoints using the PP population
2. Re-analysis of the non-inferiority endpoint using the non-parametric Mann–Whitney U test to account for potential non-normality of NRS pain scores
3. Re-analysis of the HDP incidence endpoint using Fisher’s exact test
4. Complete-case analysis of primary outcomes without imputation of missing data
5. Outlier sensitivity analysis: outliers of the total time interval from nerve block completion to PACU discharge (defined as values outside mean ± 3 standard deviations) will be excluded, and the co-primary endpoints will be re-analyzed to verify the stability of the results
6. Stratified subgroup analysis: if a significant intergroup difference in the total time interval is detected, subgroup analysis stratified by the median of the time interval will be performed for the co-primary endpoints, to explore the influence of this factor on the study results

##### Safety analysis

- The incidence of all adverse events and serious adverse events will be tabulated by treatment group, with classification by system organ class and severity (mild, moderate, severe).
- The causal relationship between each adverse event and the study intervention will be assessed by the principal investigator and the Data Monitoring Committee (DMC).
- The time to onset, duration, treatment measures, and final outcome of all adverse events will be documented and summarized.

### Data monitoring committee

An independent Data Monitoring Committee (DMC) was established to monitor trial safety, data quality and feasibility, providing objective advisory opinions on trial continuation or termination. The DMC operates independently of the study sponsor and investigators with no conflicts of interest.

1 Composition

The DMC consists of 3 independent experts:

- 1 senior anesthesiologist specializing in regional anesthesia
- 1 biostatistician with clinical trial experience
- 1 clinical ethicist

All members sign a conflict-of-interest disclosure form before joining. Any new conflicts arising during the trial must be reported immediately.

2 Core responsibilities

- *Safety monitoring*: Review all adverse events (AEs) and serious adverse events (SAEs), assess their causal relationship with the study intervention, and identify unexpected safety signals
- *Data quality supervision*: Evaluate data completeness, protocol compliance, and the integrity of blinding procedures
- *Feasibility assessment*: Monitor recruitment rates and dropout rates, provide recommendations for adjusting trial strategies if needed
- *Emergency decisions*: Recommend emergency unblinding only when essential for life-saving treatment, or recommend early trial termination if unacceptable safety risks are identified

3 Meeting and reporting

- Regular meetings are held every 3 months during enrollment, with an interim meeting at 50% enrollment (50 subjects)
- Emergency meetings are convened within 24 h of notification of a serious adverse event
- All DMC recommendations are submitted in writing to the principal investigator and institutional ethics committee, with detailed meeting minutes archived for 5 years after trial completion

4 Confidentiality

All DMC members sign a confidentiality agreement. They only access de-identified, aggregated data labeled as “Group A” and “Group B” to maintain blinding. No trial data or discussions may be disclosed to third parties until the results are formally published.

- Harms

All adverse events (AEs) occurring from the time of signing the informed consent form until the completion of the 3-day postoperative follow-up will be recorded and reported, regardless of their causal relationship with the study intervention.

5 Definition and classification

*Adverse events (AEs)*: Any unfavorable medical occurrence in a study subject, including but not limited to:

- Local anesthetic-related: Local anesthetic toxicity, allergic reaction, injection site pain, hematoma
- Nerve-related: Transient paresthesia, persistent sensory or motor deficit
- Respiratory-related: Hoarseness, Horner syndrome, dyspnea, hypoxemia
- General: Nausea, vomiting, hypotension, bradycardia, postoperative dizziness

*Serious adverse events (SAEs)*: Any AE that results in death, life-threatening condition, permanent disability, prolonged hospitalization, or requires emergency medical intervention. For this trial, special attention will be paid to severe respiratory distress requiring non-invasive or invasive ventilation, and permanent neurological injury.

6 Causal relationship assessment

Each AE will be assessed for its causal relationship with the study intervention by the principal investigator, classified as definitely related, probably related, possibly related, probably unrelated, or definitely unrelated. Only AEs classified as definitely or probably related to the study intervention will be considered treatment-related.

7 Reporting and management

- All AEs will be recorded in detail in the case report form (CRF), including onset time, severity, duration, treatment measures, and final outcome.
- All SAEs must be reported to the institutional ethics committee and the Data Monitoring Committee (DMC) within 24 h of occurrence. A written follow-up report will be submitted within 7 days with detailed information on the event and its management.
- Subjects experiencing any AE will receive appropriate medical treatment and close follow-up until the event resolves or stabilizes.

## Discussion

Since Urmey first described the 100% incidence of HDP following ISB in 1991, this complication has attracted intense clinical attention. HDP explains the transient chest tightness and dyspnea experienced by many patients after ISB, and in rare cases, can result in persistent HDP (29–34).

Unilateral HDP causes a 20 to 40% reduction in forced vital capacity (FVC) and forced expiratory volume in 1 s (FEV₁) (18, 35). Consequently, HDP must be strictly avoided in patients with preexisting pulmonary impairment, including those with moderate to severe chronic obstructive pulmonary disease (COPD), severe asthma, restrictive ventilatory disorders, multiple rib fractures, pneumothorax, persistent hypoxemia, morbid obesity, pregnancy, and advanced age (36–38). When HDP occurs, mild cases may require increased oxygen supplementation, while severe cases necessitate mechanical ventilation to maintain adequate oxygenation.

Researchers have long endeavored to reduce the incidence of HDP. Modifications to ISB include extrafascial injection, low-volume administration, and reduced local anesthetic concentrations (38–40). Alternative techniques targeting sites more distant from the phrenic nerve have also been developed, including superior trunk block, combined suprascapular and axillary nerve block, high thoracic erector spinae plane block, supraclavicular brachial plexus block, and costoclavicular block (21, 41). However, none of these approaches have achieved a 0% incidence of HDP.

A 0% incidence of HDP represents a critical unmet clinical need. Patients with impaired pulmonary function stand to benefit the most from regional anesthesia techniques, as they cannot tolerate the respiratory risks associated with general anesthesia and endotracheal intubation. Regional anesthesia is often the only viable anesthetic option for these vulnerable patients. However, even a 0.01% risk of HDP can result in endotracheal intubation and intensive care unit (ICU) admission for these patients, a scenario that clinicians strive to avoid at all costs. Therefore, there is an urgent need to investigate techniques that may provide reliable surgical analgesia while minimizing the risk of HDP, to improve the safety of surgery in this high-risk population (21).

In our preliminary experience, subparaneural injection of 2 mL 0.375% ropivacaine at the bifurcation of the brachial plexus upper trunk achieved a 0% incidence of HDP in 11 patients. Therefore, we designed this study to investigate the non-inferior analgesic efficacy and potential superior diaphragmatic safety of this 2 mL regimen compared with the standard 5 mL regimen.

This prospective randomized controlled trial protocol has several foreseeable limitations that should be acknowledged. First, this is a single-center trial conducted at a high-volume orthopedic center in China, which may limit the generalizability of the findings to medical centers with different surgical workflows, operator experience levels, or patient baseline characteristics. To mitigate this, we have developed highly standardized operating procedures for all core links of the trial, and plan to conduct a multi-center study to validate our findings after the completion of this trial.

Second, we excluded patients with preexisting obstructive ventilatory dysfunction (FEV1/FVC < 70%) to ensure the homogeneity of baseline diaphragmatic and pulmonary function, which means the results cannot be directly extrapolated to patients with underlying pulmonary impairment, the population that would benefit most from this diaphragm-sparing nerve block technique. This trial is designed as a foundational study in a low-risk population, and a subsequent dedicated trial focusing on patients with pulmonary dysfunction is planned to verify the safety and efficacy of this regimen in high-risk populations.
